# A review on: *Interdisciplinary Practices in Academia: Writing, Teaching and Assessment*

**DOI:** 10.3389/fpsyg.2026.1814476

**Published:** 2026-09-25

**Authors:** Yuehai Xiao, Xiao Peng, Li Sun

**Affiliations:** 1 Department of English, Guangzhou University, Guangzhou, Guangdong Province, China; 2 Liberal Arts College, Hunan Normal University, Changsha, Hunan Province, China; 3 School of Foreign Languages, Wenzhou University of Technology, Wenzhou, Zhejiang Province, China

**Keywords:** academic writing, cultural differences, EAP, ESP, inclusive EAP pedagogy, interdisciplinarity

## Abstract

The monolingual model does not reflect the multilingual realities of today and research across the cognitive, social and linguistic domains is still limited. In addition, there is an urgent need for a more inclusive English for Academic Purposes (EAP) pedagogy to address the heterogeneity of English academic writing across national, cultural and disciplinary contexts. These gaps are addressed in the edited volume, *Interdisciplinary Practices in Academia: Writing, Teaching and Assessment*, edited by Buckingham, Dong and Jiang, which investigates how interdisciplinarity affects the teaching and assessment of academic writing in different cultural and institutional contexts. The review critically evaluates how the volume not only examines the challenges and opportunities of this transition, but also points out its limitations for future research. The main contribution of the volume is a systematic mapping of the impact of interdisciplinarity on the linguistic and cultural dimensions of academic writing and a call for EAP/ESP research to break away from discipline-bound frameworks. Its practical orientation will appeal to EAP practitioners, administrators and researchers. Its global perspective demonstrates how local policies and cultural values influence interdisciplinary practices. Some chapters contain practical advice for classroom implementation. But the collection would have been stronger with focused attention on the assessment of interdisciplinary writing, novice writer development, and technology-mediated collaboration.

The wider epistemic shift toward integration across disciplinary boundaries is exemplified by the increasing number of interdisciplinary degree programs, research projects and scholarly publications in contemporary higher education (Huutoniemi et al., 2010). Such a trend has profound implications for English for Academic Purposes (EAP) and English for Specific Purposes (ESP), two fields that have traditionally focused on disciplinary-specific discourse analysis (Dong and Buckingham, 2022; Hu and Zhang, 2025). In the meantime, the linguistic paradigm of monolingualism has been subjected to strong criticism (Xiao and Zhao, 2022). To successfully address the heterogeneity of English academic writing across national, cultural and disciplinary contexts, EAP pedagogy needs to be more inclusive (Bakogiannis et al., 2024). Moreover, the cognitive, social and linguistic perspectives of different academic disciplinary domains are still insufficiently studied (Golden et al., 2021).

Addressing these gaps, the edited volume *Interdisciplinary Practices in Academia: Writing, Teaching and Assessment* arrives timely in higher education, when interdisciplinary approaches are attracting increasing interest from universities, research funders and policy makers (van Helden et al., 2024). These developments pose important questions for EAP and ESP practitioners on how to prepare students for academic communication and knowledge-making in increasingly dynamic academic contexts (Hu and Zhang, 2025). The book’s main contribution is its systematic documentation of the ways in which interdisciplinarity is reshaping the linguistic and cultural face of academic writing. Thus, EAP/ESP research must move beyond disciplinary-specific frameworks and address these emergent hybridity.

The volume’s interdisciplinary orientation builds on the existing literature on academic discourse studies on the ways academic writing is shaped by disciplinary cultures and global publishing pressures (see Xiao, 2011). More recently, the transnational turn in academic literacy scholarship has highlighted the challenges and opportunities of writing across linguistic, national and disciplinary borders, demonstrating the need for EAP pedagogies that are responsive to the increasing mobility of students, scholars and knowledge itself (Xiao et al., 2024). Buckingham, Dong, and Jiang’s volume extends these conversations by providing a grounded, globally oriented investigation into how interdisciplinarity is discursively enacted and pedagogically negotiated in different institutional contexts, contributing to an emerging body of work that moves beyond discipline-bound frameworks toward more integrated, transnationally informed understandings of the teaching and assessment of academic writing.

Buckingham and Dong’s Introduction provides the coherence for this edited volume, organizing the collection around three connected lenses: text genres and linguistic features; pedagogy and assessment; and the conceptualization of academic programs. This structure is systematically worked through in Part I (discourse-analytic studies) and Part II (pedagogical and institutional case studies). Each chapter offers a different empirical perspective on the volume, but there are thematic threads repeated across the chapters: the negotiation of disciplinary epistemologies, the challenges of interdisciplinary communication, and the centrality of genre awareness in the development of interdisciplinary literacy. This editorial architecture enables the collection to operate as a coherent scholarly intervention, cumulating toward a common argument that interdisciplinarity is discursively constructed, pedagogically enacted and institutionally embedded in diverse global contexts.

The editors have carefully arranged the volume into three integrated sections. Part I deals with the linguistic characteristics and rhetorical resources of interdisciplinary written texts and provides empirical analyzes of evaluative adjectives, stance markers and citation practices across disciplinary boundaries. Part II focuses on pedagogical issues, such as degree program structure, development of academic literacy courses and language proficiency assessment. Part III discusses the conceptualization and incorporation of interdisciplinarity into academic programs, including institutional policy and curriculum design. This three-part structure allows the volume to move from micro-level textual analysis to meso-level pedagogical innovations to macro-level academic program planning, although the connections between these levels of analysis are not always fully elaborated.

One of the volume’s major strengths is its global scope. Contributors draw on empirical data from Sri Lanka, Canada, Kenya, China and the United Arab Emirates, pointing to the increasing importance of inclusive pedagogies and practices in EAP in higher education contexts (Bakogiannis et al., 2024). This geographical diversity is not just cosmetic but shows how local educational policies, funding structures and cultural attitudes toward disciplinarity inform interdisciplinary practices. For example, the case studies from the Global South question the assumption that interdisciplinary work is primarily a concern of Western research universities.

## Pedagogical innovations and practical implications

Several chapters are useful for practitioners to learn to take an interdisciplinary approach to their teaching contexts. Davies, in Chapter 5, offers a detailed case study of a graduate-level EAP course at Duke Kunshan University in China, which includes an interdisciplinary oral communication component on audience awareness and cross-disciplinary dialog, and a discipline-specific academic writing component on genre conventions. This course design is a practical one that validates the students’ needs for scholarly communication through speaking and writing in their own academic community and across disciplines. Davies’s discussion of the challenges of facilitating interdisciplinary collaboration is particularly helpful for practitioners.

Likewise, Mahawattha, Rassool and Correy (Chapter 6) describe a curriculum development project through collaboration between EAP and science lecturers at a university in Sri Lanka and reveal the importance of time commitment and mutual respect as the key to effective interdisciplinary partnership. Their findings corroborate prior studies that successful interdisciplinary collaboration requires institutional requirements along with time investment and opportunities for interdisciplinary curricula development (Van den Beemt et al., 2020). The chapter’s focus on the affective and relational dimensions of interdisciplinary work complements the more technical analyzes provided elsewhere in the volume.

Bedeker and Gaye (Chapter 7) suggest a course in writing for UAE Health Sciences students, developed using ESP principles, and built around two complementary conceptual frameworks: a threshold concept approach and a Teaching Learning Cycle based on Systemic Functional Linguistics. The findings are significant because they address differential student outcomes . High achieving students performed consistently across conditions regardless of the interdisciplinary components, while lower achieving students performed significantly better when explicit scaffolding was given for academic literacy tasks. This trend suggests that interdisciplinary pedagogies and genre-based linguistic support may constitute an intervention that promotes equity by offering equal access to disciplinary knowledge to all learners. However, this claim needs to be handled with caution: the sample size and methodology of the chapter limit the generalizability of its findings, and research on inclusive EAP pedagogy stresses decolonizing the curriculum and collaboration between EAP and content teachers (Bakogiannis et al., 2024). In general, these findings caution us against expecting one curricular intervention to overcome structural inequalities.

In Kenyan private universities, the institutionalization of interdisciplinary approaches has been incorporated into institutional missions of teaching, research, and community outreach (Kinuthia, Chapter 10). The chapter additionally emphasizes the increasing tendency of employers’ preference for graduates from interdisciplinary programs, which is consistent with previous studies that found that interdisciplinary curriculum may enhance the employability of graduates, while employers value the transferable skills of graduates (Akhtar et al., 2024; García-Álvarez et al., 2022). However, Kinuthia also documents the gap between institutional descriptions and what happens in practice, which is a persistent problem. He notes that successful interdisciplinary curricula require cooperative efforts across disciplines and university supports that enable interdisciplinary curricula, teaching, and research.

## Assessment challenges and methodological limitations

Nonetheless, although the question of assessment is the subject of several chapters, it is not developed as a sustained analytical thread throughout the volume. In Chapter 9, Altiparmak presents an innovative methodological approach to the study of Aviation English speaking testing from an interdisciplinary perspective, linking the research on speech disfluency in psycholinguistics and oral fluency testing in language testing. The study reveals three important findings: (a) filled pauses and prolongations are the most common disfluency types among the Turkish ESL speakers; (b) question-answering tasks induce more disfluency production than oral summary tasks; and (c) there is no consistent relationship between examiner fluency ratings and objective disfluency frequency rates. These findings have important implications for rater training and task design in high-stakes language assessment, but the highly specialized testing context of the chapter limits its direct applicability to broader interdisciplinary writing assessment.

This limitation is indicative of a more general gap in the volume. Schembri’s chapter on graded dissertations and Altiparmak’s work on language tests are both valuable, but a chapter on the daunting difficulties of evaluating interdisciplinary writing would have strengthened the collection. Recent scholarship has highlighted the challenges of developing valid and reliable measures of interdisciplinary student work, considering the need to assess students’ ability to integrate multiple disciplinary perspectives while preserving the differences in rhetoric, theory, and method across academic disciplines (Boix Mansilla et al., 2009).

The Targeted Assessment Rubric for Interdisciplinary Writing (Boix Mansilla et al., 2009) contains four dimensions of assessment: purposefulness, disciplinary grounding, integration, and critical awareness. But it was recognized that this requires adaptation to specific disciplinary combinations and institutional settings. The book does not address the dimensions of evaluation and therefore does not yet connect the textual and pedagogical analyzes with practical assessment tools.

Moreover, the corpus-based studies in the volume, while methodologically sound, are primarily focused on published expert texts or successful student work. The pedagogical value of the linguistic analyzes is limited by an emphasis on polished products, not the work of novice writers. Longitudinal studies that investigated how novice writers manage the rhetorical and lexical demands of interdisciplinary genres, such as how they struggle to begin, make strategic compromises, and make the gradual approximations characteristic of authentic learning processes, would benefit interdisciplinary writing development research.

The book raises issues of power and equity in interdisciplinary work but could go further. Which disciplinary languages or ways of knowing are dominant in interdisciplinary spaces? How do researchers in traditionally “softer” or less powerful disciplines react to the writing demands of “harder” or more prestigious partners? How do institutional reward structures, particularly publication metrics and grant funding criteria, shape the possibilities for equitable interdisciplinary partnership? Kalan’s Chapter 1 raises these questions through the experiences of multilingual writers, and the global examples hint at local power dynamics, but future work needs to be more directly focused on this area.

Recent scholarship on inclusive EAP pedagogy has emphasized the necessity of creating inclusive learning environments through addressing linguistic and cultural stereotypes, promoting reflexive teaching, decolonizing the curriculum and advancing equity and social justice in EAP practice (Bakogiannis et al., 2024). The contributors to the volume describe diverse learner populations. Less differentiated attention is paid to the different challenges faced by undergraduate versus graduate students, or learners from different linguistic and cultural backgrounds, than the complexity of these populations warrants. Recent work on construct validity in multilingual assessment has been driven by the need to develop fairer and more valid assessments that more accurately reflect the competencies of multilingual learners (Gantefort et al., 2025).

Another limitation is the relative lack of attention to the role of digital resources in facilitating interdisciplinary collaboration in the volume. As collaborative writing platforms, digital humanities methodologies and research tools are increasingly popular, we need to pay attention to technology-mediated interdisciplinary work. The use of digital platforms like Google Docs for collaborative writing and project work has increased (Jung et al., 2017). However, limited attention has been paid to the use of these platforms in interdisciplinary academic writing within EAP/ESP contexts.

## Conclusion

Overall, the volume’s scholarly contribution is hampered by a number of underdeveloped areas which together limit its capacity to fully theorize and operationalize interdisciplinarity in EAP/ESP contexts. Together, these limitations do not lessen the volume’s substantial contributions, but they do suggest a tendency to privilege breadth of coverage over depth of critical engagement. In future research closing these gaps, by focusing more closely on linguistic detail, developing interdisciplinary assessment models, creating inclusive pedagogical frameworks that recognize learner heterogeneity and equity and critically interrogating the role of technology, would greatly move forward the agenda this volume ambitiously sets in motion.

Despite these limitations, *Interdisciplinary Practices in Academia: Writing, Teaching and Assessment* makes an important contribution to EAP/ESP scholarship. The volume’s main contribution is to show that interdisciplinarity is not a marginal concern for language education, but a central phenomenon that requires a fundamental rethinking of established pedagogical and research frameworks. The editors combine textual analysis, pedagogical case studies, and institutional perspectives to set the stage for future research.

The practical orientation of the book will make it valuable to several groups of readers. Chapters by Davies and Mahawattha et al. provide concrete models of curriculum design and faculty collaboration for EAP practitioners. Kinuthia’s discussion of challenges in institutional implementation will be useful for program administrators. In the corpus studies, researchers will find methodological templates for the study of interdisciplinary linguistic features, or will undertake theoretical investigations into the ideological dimensions of interdisciplinary discourse.

For the EAP/ESP community, perhaps the volume’s greatest contribution is its implicit argument that the field must move beyond its traditional focus on disciplinary-specific genres to develop frameworks for teaching and researching hybrid, boundary-crossing forms of academic communication. As Hu and Zhang (2025) have observed, EAP research is saturated in traditional areas, such as move-step analysis and lexical bundle identification. Against this backdrop, the linguistic and rhetorical features of interdisciplinary genres may be an underexplored area.

The edited volume makes significant progress in tackling the challenges and opportunities of the rise of interdisciplinarity in the teaching and assessment of academic writing. Some directions for future research might be its limitations, particularly concerning assessment, the analysis of the work of novice writers, and the integration of technology. The book makes a strong case for interdisciplinarity to be at the heart of EAP/ESP research agendas rather than on the margins. This volume maps the complex landscape of interdisciplinary academic writing and provides the EAP/ESP community with tools to better prepare future scholars and professionals to work, write, and create across traditional disciplinary boundaries.
